# How nurses support family caregivers in the complex context of end-of-life home care: a qualitative study

**DOI:** 10.1186/s12904-021-00854-8

**Published:** 2021-10-18

**Authors:** Yvonne N. Becqué, Judith A. C. Rietjens, Agnes van der Heide, Erica Witkamp

**Affiliations:** 1 Research Centre Innovations in Care, Rotterdam University of Applied Sciences, Rochussenstraat 198, P.O. Box 25035, 3001 HA Rotterdam, the Netherlands; 2 Department of Public Health, Erasmus University Medical Center Rotterdam, Dr. Molewaterplein 40, P.O. Box 2040, 3000 CA Rotterdam, the Netherlands

**Keywords:** End-of-life care, Family caregivers, Home care, Nurses, Support

## Abstract

**Background:**

Family caregivers are crucial in providing end-of-life care at home. Without their care, it would be difficult for many patients to die at home. In addition to providing care, family caregivers also need support for themselves. Nurses could play an important role in supporting family caregivers, but little is known about if and how they do so. The aim of this study is to explore how nurses currently approach and support family caregivers in end-of-life home care and which factors influence their support of family caregivers.

**Methods:**

Data were collected using semi-structured interviews with 14 nurses from nine home care organisations in the Netherlands, in 2018. Interviews were audio-taped, transcribed verbatim and analysed using a thematic analysis approach.

**Results:**

We identified two underlying nursing perspectives on supporting family caregivers: an instrumental perspective (seeing family caregivers mostly as collaborative partners in care) and a relational perspective (seeing family caregivers as both providing and needing support). All the interviewed nurses stated that they pay attention to family caregivers’ needs. The activities mentioned most often were: identification of support needs, practical education, support in decision-making about the patient’s treatment, emotional support, and organising respite care, such as night care, to relieve the family caregiver. The provision of support is usually based on intuition and experience, rather than on a systematic approach. Besides, nurses reported different factors at the individual, organisational and societal levels that influenced their support of family caregivers, such as their knowledge and experience, the way in which care is organised, and laws and regulations.

**Conclusions:**

Nurses tend to address family caregivers’ needs, but such care was affected by various factors at different levels. There is a risk that nursing support does not meet family caregivers’ needs. A more reflective approach is needed and evidence-based needs assessment tools may help nurses to systematically assess family caregivers’ needs and to provide appropriate support.

## Background

Most people prefer to spend their last phase of life at home [1, 2], which is in most cases not possible without the help of family caregivers [3]. Family caregivers are defined as ‘individuals who provide any physical, emotional, and instrumental support and assistance to individuals with life-limiting illness that they view as family members’ [4].

Family caregivers are often intensively involved in the care for a relative who is in the last phase of life [5]. In the United Kingdom, family caregivers of patients with advanced cancer were found to provide care a median of 69 h per week in the final 3 months of life [3]. Although caring for a relative might be rewarding [5, 6], being a family caregiver also involves challenges. They have to cope with the impending loss of a family member and providing care in itself can be a source of stress [7]. Many family caregivers are insufficiently prepared to cope with a situation where they need to take responsibility for the person’s physical and emotional care [8, 9]. As a result, they are prone to emotional, physical, social and financial distress [10–12]. Therefore, the position of family caregivers can be seen as ‘providing support’ but also ‘needing support’ [13]. This dual position is recognised in the EAPC White paper, which presents ten core competences in palliative care [14]. Health care professionals should not only be able to recognise and support family caregivers in their tasks as caregivers but should also provide them psychological and emotional support separate from the patients.

Home care nurses are often responsible for decisions about the amount of end-of-life home care which patients and family caregivers receive [15]. In the Netherlands, this responsibility of nurses is laid down in the Healthcare Insurance Act since 2015 [16]. Therefore home care nurses have a unique position to assess and to address family caregivers’ needs. However, the literature shows that home care for patients with an advanced illness is not yet focused on family caregivers [1, 9, 17, 18].

Little is known about the extent to which nurses acknowledge the dual position of family caregivers and if and how they currently fulfil a supportive role with respect to family caregivers in end-of-life care. The objectives of this study were to explore: 1) how nurses currently approach and support family caregivers in end-of-life home care; and 2) which factors influence their support of family caregivers.

## Methods

### Sample

This interview study was part of a wider study on end-of-life support of family caregivers in which ten home-care organisations in the southwest region of the Netherlands participated. The participating organisations were located in demographically different areas (cities and rural areas) and had varying organisational structures. In some organisations, palliative care is part of regular home care, but others only provide palliative care. The managers of these home care organisations were asked for permission for the interviews and were requested to invite nurses to take part. Nurses were eligible to participate if they were involved in the care of patients in their last phase of life. Managers selected nurses who met the inclusion criteria and had the time and willingness to participate.

### Data collection

A semi-structured topic guide, developed by the research team (all authors), was used that included topics on the nurses’ vision on family caregivers’ roles, their experience of nursing support for family caregivers, and facilitators and barriers for supporting family caregivers. See Table 1. The first author (YB) performed the interviews between March and August 2018. A pilot interview demonstrated the usefulness of the interview guide and resulted in the refinement of one topic. This pilot interview was included into the dataset. Prior to the interviews, all participants were informed about the study and asked for their consent and permission to record. Interviews were held as joint interviews when this was what the nurses themselves preferred. Literature suggests that joint interviewing gives participants the opportunity to support and prompt each other and can help explicate what is often tactic knowledge [19]. The interviews were held at the participants’ home care organisation and lasted an average of 52 min (ranging between 44 and 63 min).

**Table 1:** Topic guide

- Vision on family caregivers’ role in end-of-life care at home	
- Vision on the nurses’ role in end-of-life care at home	
- Assessment of the support needs of family caregivers	
- Supportive interventions for family caregivers provided by nurses and other disciplines	
- The basis on which they choose interventions	
- Registration of supportive interventions in the care plan and nursing file	
- Facilitators and barriers of family caregivers’ support	
- Chances/opportunities to support family caregivers in end-of-life care at home	

### Analysis

Audio-recordings were transcribed verbatim by an external party, checked and anonymised. Then they were entered into ATLAS.ti (version 8.4), a software program that facilitates qualitative data management and analysis. The principle of thematic analysis guided an iterative and reflective process of data collection and analysis [20]. First we read the transcripts in detail to become familiar with the nurses’ stories. Following line by line scrutiny of the transcripts, we coded the data detailing inductively codes by marking similar phrases or words from the nurses’ narratives. Coding was conducted separately by two authors (YB, EW), and the coding was subsequently compared and discussed until consensus was reached. Codes were grouped together and refined into categories. Codes referring to nursing interventions were refined into the following categories derived from previous intervention classifications [21, 22]: educational support, support in decision-making, emotional support and organising respite care. Codes referring influencing factors were ordered following the five systems of ‘The framework for complexity in palliative care’ [23]: the microsystem (person), chronosystem (dynamic influences of time), mesosytem (interactions with family and health professionals), exosystem (palliative care services/systems) and macrosystem (societal influences). The interpretations and final themes of the data were discussed within the research team. Post-interview field notes and notes from the data analysis enhanced the reflective process.

The COnsolidated criteria for REporting Qualitative research (COREQ) Checklist was used as underlying structure of this article.

### Ethical considerations

Under Dutch law this study is exempt from approval by a research ethics committee because it did not involve patients, family caregivers, interventions or burdening procedures [24].

Participants were informed about the study and aims and verbal informed consent was obtained from all participants before the start of the interview. At the end of the interview, the researcher summarised the main issues discussed in the interview to allow the participant to verify and clarify any misconceptions or add further information. The transcripts of the interviews were anonymised.

## Results

This results section starts with a description of the characteristics of the interviewed nurses, followed by an explanation of two nursing perspectives on family caregiving. Then current practices of provision of nursing support and the influencing factors will be addressed.

Fourteen nurses, from nine organisations, were invited for the interviews. All nurses were willing to participate and were interviewed. One organisation could not participate because it was in the middle of a reorganisation. As Table 2 shows, eight individual and three joint interviews were carried out. Twelve of the 14 interviewed nurses were female. Nurses’ ages ranged from 23 to 59 (mean of 45). They were all registered nurses, with most having had additional training in palliative care. Three nurses had less than 5 years of healthcare experience, two between 10 and 20 years, eight more than 20 years and one unknown. Five nurses worked in a (specialised) home care organisation or team that only provided palliative care.

**Table 2:** Characteristics of the 14 nurses from the nine home care organisations

Participant number	Home care organisation	Specialised pall. care	Gender of nurse	Age of the nurse	Type of interview
P01	HCO1	Yes	Male	50–59	Duo with P02
P02	HCO1	Yes	Female	50–59	Duo with P01
P03	HCO2	No	Female	30–39	Duo with P04
P04	HCO2	No	Female	40–49	Duo with P03
P05	HCO3	No	Female	20–29	Individual
P06	HCO4	No	Female	20–29	Individual
P07	HCO4	No	Female	40–49	Individual
P08	HCO5	No	Female	40–49	Individual
P09	HCO5	Yes	Female	50–59	Individual
P10	HCO6	No	Female	50–59	Duo with P11
P11	HCO7	No	Female	50–59	Duo with P10
P12	HCO8	Yes	Female	20–29	Individual
P13	HCO8	Yes	Female	30–39	Individual
P14	HCO9	No	Male	50–59	Individual

### Nursing perspectives

How nurses support family caregivers depends on the values and beliefs of nurses.

Two nursing perspectives emerged from our inductive analysis: the ‘instrumental perspective’ and the ‘relational perspective’.

Nurses with the instrumental perspective focus more on the patient than on the family caregivers. They view family caregivers predominantly in relation to the patient and they see them as collaborative partner in the care of the patient.

*“The client is of course the most important person, everything revolves around the client's wish, but the family caregiver is continuously with the client. I give a lot of advice on how to deal with certain things they encounter, for example pain relief.” (P09)*

*“The family caregiver as a ‘co-worker’ is the ideal. For me, that means the family caregiver is very involved and wants to help, for example, with turning the client over or washing the client, or the family caregiver makes sure that everything needed is in place, such as the medical equipment and medication. The family caregiver as co-client shouldn’t distract from the person you are actually there for and that is the client who is dying.” (P07)*

Nurses adopting the instrumental perspective underline the importance of professional knowledge and base their interventions to support family caregivers on guidelines and protocols. They think it is important to educate family caregivers about symptom management and the process of going through an advanced illness and dying.

In the relational perspective, nurses see the patient and the family caregiver as inextricably linked and they acknowledge that family caregivers may have a dual position:

*“I make people aware of the difference between those two roles [partner and carer]. I find that really essential. I really want to keep them apart. My experience is that family caregivers are then more likely to go through the emotional experience during the dying phase and to say farewell. If you're just busy caring, caring, caring then you're not so emotionally involved in taking leave of someone.” (P14)*

Intuition and experience play a major role for nurses who engage with the relational perspective, and for whom the emotional support of family caregivers is an essential aspect of the care they provide.

*“I’ve actually been involved with it [death and dying] since I was a kid. I’ve seen so many mourning processes. So yes, you learn a lot from that, as a human being it is so enriching. And then you also see it as a nurse, and you recognise all that. It's not like I have to read about it or think about it. It comes naturally.”(P01)*

Nurses with this perspective criticise the healthcare system for being too “clinical” and too patient-oriented.

### Nursing support

Nurses mentioned a variety of activities to support family caregivers in end-of-life care. We identified five main types of support:

1. Identification of needs,
2. Practical education,
3. Support in decision-making about place of death and treatment,
4. Emotional support,
5. Organising respite care.

#### Identification of needs

Participants reported that they identify the needs of family caregivers on admission and more informally during patient’s care, while having coffee or on the doorstep when leaving. A few nurses make a specific appointment with the family caregiver to discuss their needs.

They explained that this assessment is not formally structured but rather informal, by asking open questions such as ‘how are you doing?’ and ‘what do you need?’ Some nurses found it difficult to identify the needs of family caregivers. They mentioned that history taking and needs assessment are typically focused on the patient rather than the family caregiver. According to some nurses, an assessment or interview tool to identify the needs of family caregivers would be useful.

*“A scan or another tool at the start, to assess how someone is doing. And maybe you can repeat that during an evaluation. [...] So that you also get insight into how the family caregiver is doing and what the caregiver might need, independently of the patient.” (P10)*

*“What's important for those relatives to discuss? I think we're doing it intuitively, but maybe there are standard issues that are not mentioned or discussed. ” (P06)*

In addition to identifying needs, some participants told how they informally assess family caregivers’ burden, by using open questions and being alert to signs of burden. One nurse uses the Caregiver Strain Index (CSI) to assess burden and strain. The nurse used the CSI as standard part of the care plan to gain insight into the strain on family caregivers, but merely saw it as a formality.

#### Practical education

Nurses spoke of how they teach and instruct family caregivers to perform practical care tasks, such as washing their relative, turning them in bed or giving medicines. They also discuss which symptoms patients may experience and how family caregivers can support patients practically.

The nurses also mentioned that they provide educational support by discussing with family caregivers what to expect regarding their relative’s illness and dying process.

*“They just really didn't know how the dying process goes. And if you explain that step by step, what can be expected, then you notice that they were pleased because now they really were prepared. The most difficult part remains, but they were prepared and I saw that that was really good for them.”(P03)*

#### Support in decision-making

In order to support family caregivers in their role in making decisions with the patient, nurses reported that they explain the options, e.g. for the place of death (at home or in a hospice, for example) and the advantages and disadvantages of different places to die. The pros and cons of specific treatments and care are also be discussed by the nurses. Some nurses added that they invite the general practitioner to these conversations or family meetings.

#### Emotional support

The participants thought that a sympathetic ear is an important emotional intervention. Nurses illustrated that they listen to family caregivers’ narratives about their feelings, coping problems, hopes and family relationships. Some nurses felt that it is important to give family caregivers insight into their combined roles as caregiver and spouse/partner, and ‘taking time for yourself’.

*“If you make them aware from the beginning of the palliative phase: take time for yourself, take time for your children and friends. Of course the mourning continues but they haven't lost themselves in the role of caregiver.”(P14)*

The nurses mentioned that when they do not find themselves capable of dealing with certain emotional or existential needs of family caregivers, they refer them to other professionals, e.g. a spiritual carer.

#### Organising respite care

All the nurses interviewed emphasised the importance of organising respite care to support family caregivers. They arrange night care, extra home care, volunteer support and helping caregivers draw on their social network.

*“It’s very nice to have volunteers involved because they can really stay with someone for a couple of hours. We [nurses] come in and go away again. They can stay longer and they also ensure that they are there when the family caregiver for example wants to go out for a while, they can go for a bike ride or do some shopping, or whatever.” (P09)*

### Factors influencing nursing support

Nurses indicated that various factors play a role in their approach and support of family caregivers in end-of-life care. We distinguished factors at the level of the microsystem, chronosytem, mesosystem, exosystem and macrosystem. See Table 3.

**Table 3:** Factors influencing nursing support

Microsystem (person)	Chronosystem (influences of time)	Mesosystem (interaction with family and professionals)	Exosystem (palliative care services and systems)	Macrosystem (societal influences)
- Characteristics family caregivers - Characteristics nurses	- Late referrals	- Different beliefs between nurses and caregivers - Collaboration with other professionals	- Organisation of care - Registration systems - Availability of services	- Societal and policy developments - Laws and regulations

#### Microsystem

The microsystem influencing nursing support concern the level of the person and focus on person’s needs and characteristics. The nurses mentioned how demographic and personal characteristics of the family caregivers, such as age, socio-economic status, housing situation, needs and preferences influence nursing support.

*“Socio-economic factors also play a very big role. People with medium to high incomes have very different needs. They often have a different way of life than people with a low socio-economic status, or people with an immigrant background. They have very different problems. You have to approach it very differently.” (P14)*

The nurses reported that the characteristics of the nurses themselves also play a role in how they support family caregivers. For example, age, personal and work experience, nursing perspective and competence. Some nurses reported that colleagues may have insufficient knowledge about end-of-life care; they fail to recognise problems that can arise in end-of-life care, resulting in not appropriate support of family caregivers and late referrals.

*“You have to respond to what you hear and what you see. I think that's really one of the most important things in palliative terminal care. You have to be able to anticipate the situation and not every nurse can't do that. It is quite difficult; you need to have some knowledge.” (P09)*

#### Chronosystem

Factors at the chronosystem affecting family caregivers’ support refer to changes in a person’s needs, circumstances and environment over time. Many nurses emphasised how late referrals for nursing support of family caregivers occur. The nurses explained that aspects, such as changing support needs of family caregivers, long-term burden, a rapid and unpredictable disease process, late identification of the seriousness of the palliative situation by other nurses may lead to late referrals for nursing support. They also indicated that after late referrals sometimes good support is not possible because the focus is on managing the ‘crisis’ situation.

*“I often see that home care nurses realise too late that someone is so ill. We receive the referral from a home care team, for example three days before someone dies.” (P13)*

#### Mesosystem

The meso factors that influence nursing support concern the interaction between nurses, families and other health care professionals. Participants reported the differences in how family caregivers and nurses would prefer to manage the support. Nurses said that they are sometimes not allowed to provide support because family caregivers want to do it all themselves for as long as possible or because they feel it is an intrusion into their private life.

*“There are family caregivers who are totally focused on caring. Sometimes they've been doing it themselves for a long time before we come into the picture. I think that's still the main risk group, because they're often so passionate about caring and often find it difficult to hand that task over to others. Especially this group is important to support, to demonstrate what kind of relief it can give when a professional person takes over some of the care.” (P13)*

Nurses indicated that the extent to which they have contact with other care providers, such as GPs, other nurses and physicians from the hospital, has an impact on their support of family caregivers. One participant illustrated that he had good contact with some hospital nurses and received good transfers for the support of family caregivers. While other nurses described that poor contact with other care providers and the unfamiliarity of each other’s work field led to late referral or unrealistic expectations of support. For example, one nurse said:

*“I notice at the hospital in particular that there is a real lack of knowledge of what is possible in home care. And then they [hospital nurses] easily ask for 24-hour care. The hospital's sense of security, that there is always a nurse walking around – that may not be necessary at all.”(P13)*

Many nurses mentioned that they participate in a palliative care network. This network keeps them informed about the latest knowledge about supporting possibilities and provides them short lines of contact with other care providers, such as palliative home care volunteers and palliative nurses.

#### Exosystem

The exosystem consists of services and systems which affects family caregivers’ support. For example, how nursing teams work or are structured. Some nurses experienced time pressure from the daily care schedule when the next patient is waiting for them. Nurses felt they have limited time to give attention to family caregivers, or that support is mainly given at the end of a patient visit, ‘on the doorstep’ when leaving. Task differentiation also influences which interventions nurses undertake, according to the nurses. They explained that in some home care organisations there is a division of tasks between regular home care nurses and specialised palliative care nurses. The specialised nurses provide more complex and emotional support to family caregivers. While the regular home nurses provide more physical care.

Many nurses also discussed the issue of the registration systems which focus on the patient rather than on the family caregivers. Such systems do not include a separate assessment or care plan for family caregivers, which inevitably makes care predominantly patient-centred.

*“I think there's still benefit to be got in mentioning the family caregivers separately in the care plan. To consciously take the time for them. Very often you're only dealing with the client, for example wound care. But there’s a family caregiver downstairs, and what is going on in that caregiver's head at that moment?.”(P12)*

According to the nurses, certain services to support family caregivers may be unavailable in some regions, such as hospice care and volunteer support.

#### Macrosystem

The macrosystem that influence nursing support concern the wider societal and cultural context. This could include: social developments, legislation and registration systems. Nowadays, patients and family caregivers are to a large extent expected to create their own support network and to remain independent of professional care as much as possible. The interviewed nurses indicated that some nurses and caregivers advocate this ‘self-management approach’ in end-of-life care. The participants felt however that this may be contrary to important principles in end-of-life care, such as enhancing comfort and quality of life, and may result in nurses being too reactive, rather than proactive, and failing to take action in good time.

*“Our mission is to keep them independent for as long as possible, but also to keep someone active. Care used to be about pampering. But now the elderly are expected to cooperate in getting to the healthiest possible situation and it’s about not taking over but supervising them until they can become independent again. And there’s a discrepancy. Only I think palliative care is different; then I think you should be allowed to pamper. That's the last stage of life.” (P07)*

Another important factor that was mentioned by all the nurses relates to laws and regulations. Health insurers have their own rules regarding palliative care and certain policies are laid down by law. Health insurers determine, for example, how much time nurses can take for assessments and certain forms of care, under what conditions night care can be provided and what care or support is reimbursed by the insurer. A nurse also indicated that regulations can make it more difficult to arrange respite care.

## Discussion

This article focus on the question: how do nurses currently approach and support family caregivers in end-of-life home care, and which factors influence their support of family caregivers? Two nursing perspectives on supporting family caregivers were identified from the nurses’ stories. Nurses provide a variety of supportive activities, predominantly based on their intuition and experience. Besides, nurses reported different factors on different levels influenced their support of family caregivers.

The interviews revealed two perspectives among nurses on the support of family caregivers: the instrumental perspective and the relational perspective. Ward-Griffin and McKeever [25] described four types of nurse-family caregiver relationships: nurse-helper, worker-worker, manager-worker and nurse-patient. Each type conceptualises the roles of the nurse and the family caregiver. The instrumental perspective we found is in line with the worker-worker role. Nurses in this worker-worker role focus primarily on the patient. They have an instrumental recognition of the family caregiver and teach them technical skills. The relational perspective shows a similarity to the nurse-patient type of relationship. In this type, nurses see the family caregiver both as a worker and as a patient in their own right. Nurses expressed concern for the health and well-being of the family caregivers in their care situation at the patient’s end of life. Our findings show two different bases on which nurses act in their relationship with family caregivers: guidelines and evidence-based knowledge; or intuition and experience. In addition, it is plausible that family caregivers also have differing perspectives. The study of Bijnsdorp, Pasman [26] show different profiles of family caregivers in end-of-life care, which reflect different support needs and experiences with caregiving. It is important that nurses reflect on their own perspective and the way this affects their behaviour towards family caregivers, and subsequently verify whether their approach meets the needs of family caregivers.

Our findings show that the identification of needs and the support of family caregivers by nurses are mainly based on intuition and experience, rather than on a systematic approach. As a result, the further support provided by the nurses may vary and this variation is merely based on nurses’ interpretation instead of family caregivers’ reported needs. Other studies also found that non-systematic support involves risks that the needs of family caregivers are not met [18, 27]. This intuitive approach may result from the lack of evidence-based strategies and instruments to assess family caregivers’ needs [28]. In situations where there is insufficient evidence and that involve complex problems, such as end-of-life care, nurses use intuition to decide on the tasks and actions to be taken [29]. However, little is known about the effects of grounding care in nurses’ intuition on family caregivers’ outcomes [30]. Some interviewed nurses recommended using tools to systematically assess family caregivers’ needs. Ewing, Grande [31] have developed an evidence-based comprehensive Carer Support Needs Assessment Tool (CSNAT) to assess family caregivers’ needs in end-of-life care. This tool can only be used within a person-centred approach, in which relatives are engaged in the care. Given that the value of using intuition is not clear and many family caregivers still experience unmet needs [32, 33], a more person-centred approach of family caregivers and further development and utilisation of such evidence-based family caregivers’ needs assessment tools and supportive, interventions may be beneficial.

Caring for the family caregiver is what the professional profiles expect nurses to do [14, 34] and according to the Dutch Health Insurance Act, they also have the possibility to indicate and arrange this [16]. However supporting family caregivers was hampered and affected not only by nurses’ perspectives on the family caregiver’s role, but also by various other practical and contextual factors. A major restriction is e.g. the administrative systems which is supported by other studies. Family caregivers do not have their own assessment and care plan separate from the patient’s care plan, and family caregivers’ assessment was often undocumented. Therefore, family caregivers remain ‘invisible’ in administrative systems and do not get the full attention they deserve [17, 35, 36].

There is a conflict in terms of the nurses’ role. This is in line with the study of Dierckx de Casterle, Goethals [37], who explored nurses’ daily practice from an ethical perspective. Contextual factors such as prevailing views and expectations, guidelines, protocols and procedures have a big impact on how nurses reasoned and practised in care situations. Nurses experience difficulties in translating all aspects of nursing care into daily practice. They are mainly guided by contextual factors rather than by the needs and well-being of patients or family caregivers [37]. Although support takes place in a complex context, nurses should reflect on their responsibilities regarding family caregivers’ support and take a position on how to care for family caregivers in end-of-life care.

### Strengths and limitations

We interviewed 14 nurses, which number may have been too limited to achieve saturation at all points. Nurses often indicated that they find it difficult to explicitly describe their nursing actions and were therefore often asked for examples.

The participating nurses were not selected at random but by the manager of the home care organisation. This may have resulted in selection bias. Participants were possibly more ‘palliative care minded’, meaning that nurses with considerable experience with and interest in palliative care were perhaps more likely to be invited to participate.

Joint interviews were held if invited nurses preferred it that way. As recommended in literature [19], this was done with nurses who knew each other well and had shared experiences. We feel that joint interviewing gave more depth to the interviews due to the interaction between the nurses and to unconscious nursing actions being made more explicit.

## Conclusions

In this study nurses acknowledged potential care needs of family caregivers, but their ability to meet these needs was affected by factors such as social influences, healthcare policy, administrative procedures, and their views on family caregivers’ dual roles. The non-systematic and informal approach in nursing support may involve a risk for an adequate response to the needs of family caregivers, which highlights the importance of the structured assessment of family caregivers’ needs. A more reflective and preventive approach is needed, using tools for needs assessment and evidence-based supportive interventions. In this context, nurses should be aware of their own perspective and preferences and of the appropriateness of a particular approach in each caregiver situation.

## Data Availability

The data of this study are stored at Rotterdam University of Applied Sciences, Research Centre Innovations in Care, the Netherlands. Data are available upon reasonable request to the corresponding author.

## Abbreviations

COREQ: COnsolidated criteria for REporting Qualitative research
CSI: Caregiver Strain Index
CSNAT: Carer Support Needs Assessment Tool
